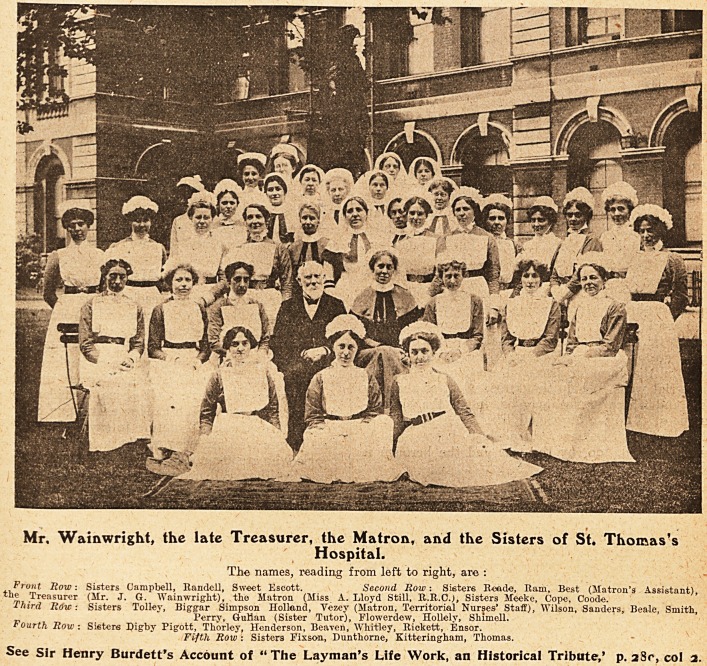# St. Thomas's Hospital. An Historic Group

**Published:** 1917-07-07

**Authors:** 


### THE MATRONS' AND SISTERS' DEPARTMENT.

### ST. THOMAS'S HOSPITAL: AN HISTORIC GROUP.

**Mr. Wainwright, the late Treasurer, the Matron, and the Sisters of St. Thomas's Hospital.**

The names, reading from left to right, are :

*Front Row*: Sisters Campbell, Randell, Sweet Escott. *Second Row*: Sisters Reade, Ram, Best (Matron's Assistant), the Treasurer (Mr. J. G. Wainwright), the Matron (Miss A. Lloyd Still, R.R.C.), Sisters Meeke, Cope, Coode. *Third Row*: Sisters Tolley, Biggar Simpson Holland, Vezey (Matron, Territorial Nurses' Staff), Wilson, Sanders, Beale, Smith, Perry, Gullan (Sister Tutor), Flowerdew, Hollely, Shimell. *Fourth Row*: Sisters Digby Pigott, Thorley, Henderson, Beaven, Whitley, Rickett, Ensor. *Fifth Row*: Sisters Fixson, Dunthorne, Kitteringham, Thomas.

**See Sir Henry Burdett's Account of "The Layman's Life Work, an Historical Tribute,' p. 28c, col 2.**

## Figures and Tables

**Figure f1:**